# An intensity-based post-processing tool for 3D instance segmentation of organelles in soft X-ray tomograms

**DOI:** 10.1371/journal.pone.0269887

**Published:** 2022-09-01

**Authors:** Angdi Li, Shuning Zhang, Valentina Loconte, Yan Liu, Axel Ekman, Garth J. Thompson, Andrej Sali, Raymond C. Stevens, Kate White, Jitin Singla, Liping Sun

**Affiliations:** 1 iHuman Institute, ShanghaiTech University, Shanghai, China; 2 School of Life Science and Technology, ShanghaiTech University, Shanghai, China; 3 University of Chinese Academy of Sciences, Beijing, China; 4 Department of Biosciences and Bioengineering, Indian Institute of Technology Roorkee, Roorkee, Uttarakhand, India; 5 Department of Biological Sciences, Bridge Institute, University of Southern California, Los Angeles, CA, United States of America; 6 Department of Anatomy, University of California San Francisco, San Francisco, CA, United States of America; 7 Molecular Biophysics and Integrated Bioimaging Division, Lawrence Berkeley National Laboratory, Berkeley, CA, United States of America; 8 California Institute for Quantitative Biosciences, Department of Bioengineering and Therapeutic Sciences, Department of Pharmaceutical Chemistry, University of California, San Francisco, San Francisco, CA, United States of America; 9 Department of Chemistry, Bridge Institute, University of Southern California, Los Angeles, CA, United States of America; Clinic for Infectious and tropical diseases, Clinical centre of Serbia, SERBIA

## Abstract

Investigating the 3D structures and rearrangements of organelles within a single cell is critical for better characterizing cellular function. Imaging approaches such as soft X-ray tomography have been widely applied to reveal a complex subcellular organization involving multiple inter-organelle interactions. However, 3D segmentation of organelle instances has been challenging despite its importance in organelle characterization. Here we propose an intensity-based post-processing tool to identify and separate organelle instances. Our tool separates sphere-like (insulin vesicle) and columnar-shaped organelle instances (mitochondrion) based on the intensity of raw tomograms, semantic segmentation masks, and organelle morphology. We validate our tool using synthetic tomograms of organelles and experimental tomograms of pancreatic *β*-cells to separate insulin vesicle and mitochondria instances. As compared to the commonly used connected regions labeling, watershed, and watershed + Gaussian filter methods, our tool results in improved accuracy in identifying organelles in the synthetic tomograms and an improved description of organelle structures in *β*-cell tomograms. In addition, under different experimental treatment conditions, significant changes in volumes and intensities of both insulin vesicle and mitochondrion are observed in our instance results, revealing their potential roles in maintaining normal *β*-cell function. Our tool is expected to be applicable for improving the instance segmentation of other images obtained from different cell types using multiple imaging modalities.

## Introduction

Subcellular architectures including organelle volume, distribution, locations, and interactions reflect various information regarding the cell state. For example, mitochondria change their localization to meet the corresponding cellular energy demands [1]. Moreover, rearrangements of insulin vesicles in pancreatic *β*-cells under different treatments reveal potential impacts in stimulating insulin secretion [2]. Furthermore, the relative localizations of insulin vesicles and mitochondria network point out the role of inter-organelle interactions during insulin secretion [3]. Thus, characterization of rearrangements of subcellular structures will facilitate our understanding of cell structure and function.

Recently a non-invasive 3D imaging technique known as soft X-ray tomography (SXT) has been developed to describe and quantify subcellular reorganization processes comprehensively [4–9]. SXT is capable of investigating the cellular architecture of intact cells in a near-to-native, hydrated, and vitrified state [10], exploiting the cells’ natural contrast at the “water window” (284–583 eV photon energy) [11], where the X-ray beam is absorbed by carbon-rich and nitrogen-rich components. For example, SXT has been used to image the mitochondria volume ratio under antifungal peptoids treatment in *C.albicans* cells [12]. In a recent study, *White et al.* applied SXT to generate three-dimensional reconstructions of whole pancreatic *β*-cells, illustrating the organelle locations and interactions [2].

Segmentation is an important step in identifying and analyzing the organization of specific organelles throughout the 3D volume of the SXT tomograms. The identification of organelles in SXT data is mainly based on two features: their morphology (e.g. spherical or elongated) and density of bioorganic components which are intrinsically connected with their molecular composition. Segmentation methods include manual segmentation, traditional algorithm segmentation like watershed, and auto-segmentation based on machine learning. To this date, the most commonly used approach is to manually segment SXT tomograms using software such as Amira-Avizo (ThermoFisher Scientific) [13], Chimera [14], Fiji [15], MITK [16]. Watershed method [17] is a traditional segmentation algorithm that relies on mathematical morphology from topological theory, dividing the spatially adjacent voxels with similar values into one label. Auto-segmentation methods train neural networks by using existing segmentation masks to predict segmentation masks on raw images. For example, *Ekman et al.* takes advantage of Convolution Neural Networks (CNNs) and annotation database to generate organelle segmentation masks on *Chromochloris zofingiensis* cell [18]. However, despite that many efforts are made on SXT organelle segmentation [17, 19, 20], especially several auto-segmentation methods [21, 22], current methods provide information of organelles, but not individual organelle instances.

Here we developed and applied an intensity-based post-processing tool to refine the segmented masks by separating organelle instances. We use SXT tomograms of pancreatic *β*-cells as a case study to characterize subcellular structures, for example, insulin vesicles and mitochondria. Our tool is based on the intensity of raw SXT tomograms, semantic segmentation mask, and prior knowledge of the organelle morphology. We first validate our method on a synthetic benchmark and compare the segmented instances with commonly used methods: connected regions labeling, watershed, and watershed + Gaussian filter methods. Then, we apply it to separate insulin vesicle and mitochondria instances from SXT tomograms of *β*-cells, and analyze organelle volumes and intensities under different conditions as well as the biological implications.

## Materials and methods

### Input data

Input data includes 24 soft X-ray tomograms and two manually segmented semantic masks (insulin vesicle and mitochondrion) for each tomogram. The tomograms are collected after 30 min treatment under three conditions, namely, 0 mM glucose (no external stimuli), 25 mM glucose, 25 mM glucose + 10 nM exendin-4 (Ex-4, a glucagon-like peptide-1 receptor agonist that enhances glucose-stimulated insulin secretion). For each condition, eight tomograms are collected. Experimental details for different treatment conditions can be found in *White et al.* [2]. The resolution of each tomogram is approx. [500, 500, 500] voxels, with each voxel having a sampling size of [35 nm, 35 nm, 35 nm]. The intensity in each voxel numerically equals to Linear Absorption Coefficient (LAC) [23] value on the current region. LAC value reflects the molecular densities of each voxel in the tomogram [U+FF0C] which is quantified by the Beer-Lambert’s law [24].

### Synthetic benchmark

Organelles are often in contact with each other in a crowded cellular environment, raising the difficulty of recognizing individual instances of organelles. To evaluate the accuracy of our tool in separating organelles, we establish two synthetic benchmarks to identify individual insulin vesicle and mitochondrion. For each type of organelle, we first manually select approx. 100 organelle instances that appear disconnected from all 24 semantic masks of this organelle. Then we construct ten synthetic datasets, each by attaching 5 randomly selected instances out of the 100 organelle instances in random positions. Each synthetic dataset includes a synthetic tomogram, a synthetic semantic mask, and a synthetic instance mask (groundtruth).

### Watershed method

Watershed is a region-based segmentation method based on the mathematical morphology [25]. It decomposes a tomogram into several catchment basins by representing voxels with higher intensities as “hills” and darker voxels as “valleys”. One major limitation of watershed method is the “over-segmentation” of noisy tomograms [26]. With the application of a Gaussian filter to denoise the tomogram, the performance of watershed method has seen some degrees of improvement [27].

## Results

We first describe the workflow of our intensity-based post-processing tool. We illustrate our approach using synthetic tomograms and *β*-cell tomograms. In both cases, the performance of our method is better than the commonly used methods of connected regions labeling [28], watershed, and watershed + Gaussian filter. Moreover, we characterize intensity and volume for the segmented insulin vesicle and mitochondria instances from *β*-cell tomograms, collected under three conditions: 0 mM glucose, 25 mM glucose, 25 mM glucose + 10 nM Ex-4; This analysis provides extra information for the subcellular structural variances under different treatments.

### Workflow

**Fig 1:**
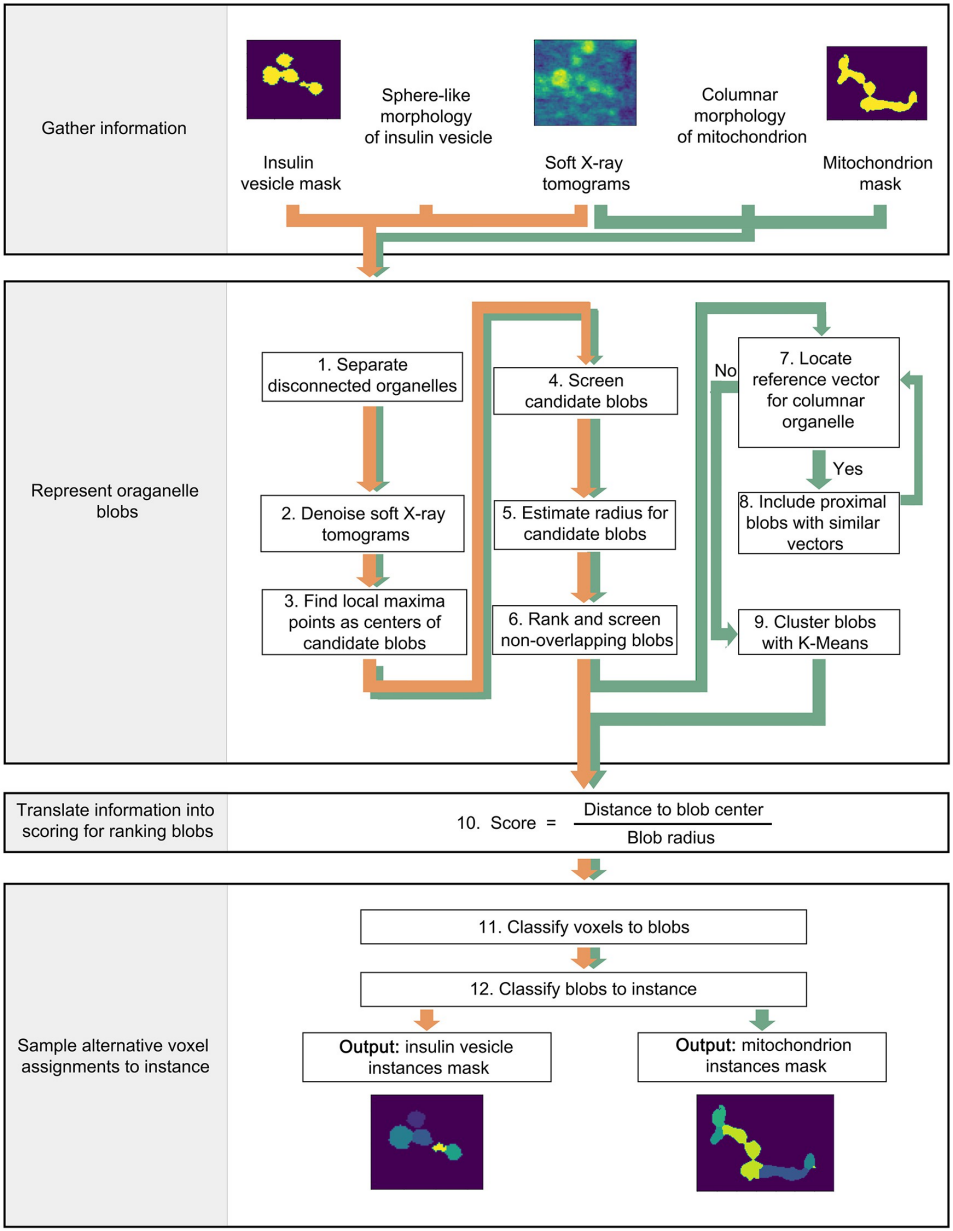
Workflow of the post-processing tool to separate insulin vesicle and mitochondria instances. Orange arrows indicate steps to separate insulin vesicle instances, while green arrows indicate steps to separate mitochondria instances. All steps are processed in 3D spaces.

Our post-processing tool is an improved blob detection method which functions by integrating information including intensity of the raw tomogram, semantic segmentation mask of the organelle, and prior knowledge of the organelle morphology (see Fig 1). The organelle morphology used here are: sphere-like shapes (i.e., insulin vesicles) [29] and columnar shapes (i.e., mitochondria) [2, 30–32]. Other typical mitochondrial shapes, such as loop and vase, which are more often seen under carbonyl cyanide m-chlorophenyl hydrazine (CCCP) exposure, are not considered here [32]. The source code of our tool is available at: https://github.com/SaliLab-SH/post_processing_tool_for_instance_segmentation_on_SXT.

**Step 1: Separate disconnected organelles:** Initially, we separate disconnected organelle regions on semantic masks into clusters of organelles. Each cluster contains one or many organelle instances. The next steps segregate each disconnected organelle cluster into individual organelle instances.
**Step 2: Denoise soft X-ray tomograms:** For each organelle cluster in the semantic mask, a rectangular box is cropped from the corresponding region in raw SXT tomograms. The box contains all voxels of the organelle cluster plus two additional layers of voxels along each edge of the box. This region is denoised using Gaussian filters. The Gaussian filter function [33] is
  $$\begin{array}{c} G_{\sigma }=\frac{1}{2\pi \sigma }e^{-\frac{x^{2}+y^{2}+z^{2}}{2\sigma^{2}}} \end{array}$$ (1)
  Here we apply a [3,3,3] Gaussian kernel to the raw tomogram. *x*, *y*, *z* are the relative coordinates to the kernel center. To maximally reserve useful information and reduce noise from the raw tomogram, we apply ten filters with *σ* ranging from 1 to 10 with 1 increment resulting in ten denoised tomograms for each organelle cluster (S1 Fig). *σ* values smaller than 1 or larger than 10 fail to provide reasonable intensity variations.
**Step 3: Find local maxima points as centers of candidate blobs:** Local maxima points are voxels whose intensity values are larger than surrounding voxels. For each organelle cluster, we apply a [3,3,3] local maxima kernel and collect local maxima points from all ten denoised tomograms as centers of candidate blobs.
**Step 4: Screen candidate blobs:** To increase the computing efficiency, centers of candidate blobs determined in step 3 are filtered out if the corresponding voxels are not located in the semantic mask.
**Step 5: Estimate radius for candidate blobs:** First, we fit a number of spheres with different radius *r* centered at the center of each candidate blob. The minimum radius *r*_*min*_ is 1.5 voxels, a threshold to restrict the minimal size of a blob. Whereas the maximum radius *r*_*max*_ is half of the diagonal length of the rectangular box determined in step 2, to include all the possible radii for the candidate blob. For each radius, the overlapping ratio is calculated between each sphere and the semantic mask by:
  $$\begin{array}{c} a_{r}=\frac{V_{m}\left(r\right)}{V_{s}\left(r\right)},r\in \left[r_{min},r_{max}\right] \end{array}$$ (2)
  *V*_*m*_(*r*) is the volume of the semantic mask containing voxels whose distance to the center of the candidate blob is smaller than *r*, *V*_*s*_(*r*) is the volume of a sphere with a radius of *r*. With the radius increases from *r*_*min*_ to *r*_*max*_, the overlapping ratio *a*_*r*_ decreases from 1 to 0 (S2A and S2B Fig). The calculation starts from *r*_*min*_ and ends when the overlapping ratio reaches 0.8, a manually adjusted threshold. The sphere used here is only to estimate the blob radius, not to represent the final blob shape.
**Step 6: Rank and screen non-overlapping blobs:** All candidate blobs are ranked based on the intensity of the center voxels in raw tomograms, determined in step 3. Then, we take a greedy strategy to screen non-overlapping blobs: 1) we first select the highest-ranked candidate blob; 2) we go through the rank list of the candidate blobs and select it if its distance to each previously selected blob is larger than the radius of the previously selected blob; 3) after screening all the candidate blobs, the final list of selected blobs will go through the following steps to obtain individual organelle instances. An example of filtered blobs is shown in S1L Fig.
**Step 7: Locate reference vector for columnar organelle:** We identify an individual mitochondria instance and its overall orientation using three blobs that are approximately aligned in a straight line (defined by the absolute value of the cosine of an adjacent angle, details in below). Using these three blobs as nucleation points, we grow mitochondria instance by adding nearby blobs. This process repeats until all the blobs are classified in mitochondria instances. We represent the overall orientation of each mitochondria instance with a reference vector ${\vec{V}}_{ref}$. To locate this reference vector, we first generate a candidate reference vector for each blob *B*_*i*_. We identify two nearest blobs $B_{i}^{1}$ and $B_{i}^{2}$ and compute two vectors $\vec{B_{i}B_{i}^{1}}$ and $\vec{B_{i}B_{i}^{2}}$, pointing from the center of blob *B*_*i*_ to the center of blobs $B_{i}^{1}$ and $B_{i}^{2}$, respectively. Then we calculate an adjacent angle *θ*_*adj*_ between these two vectors. Each blob thus has one corresponding adjacent angle. Now, if |*cos*(*θ*_*adj*_)| > *cos*(30°) (i.e. the angle is within the region [0°, 30°] or [150°, 180°]), the sum (when *θ*_*adj*_ [0°, 90°]) or subtraction (when *θ*_*adj*_ [90°, 180°]) of these two vectors is thus a candidate reference vector ${\vec{V}}_{cref}^{B_{i}}$ generated from center of blob *B*_*i*_:
  $${\vec{V}}_{cref}^{B_{i}}=\{\begin{array}{ccc} \vec{B_{i}B_{i}^{1}}+\vec{B_{i}B_{i}^{2}} & & \theta_{adj}\in \left[0^{\circ },90^{\circ }\right]\\ \vec{B_{i}B_{i}^{1}}-\vec{B_{i}B_{i}^{2}} & & \theta_{adj}\in \left[90^{\circ },180^{\circ }\right] \end{array}$$
  Among all the candidate reference vectors, the one with the maximum value of |*cos*(*θ*_*adj*_)| is assigned as ${\vec{V}}_{ref}$.
  As the reference vector has three blobs associated with it, these three blobs are assigned as one mitochondria instance label (S2C and S2D Fig). They act as nucleation of a single mitochondria instance. Then we proceed to step 8 to include proximal blobs to the instance label assigned here. We iterate between step 7 and 8 to generate as many instance labels as possible. Once we reach a point where no new reference vector can be located, either due to the number of blobs being smaller than three or the absolute value of the cosine of the adjacent angle smaller than cos(30°), we proceed to step 9 to cluster blobs with K-Means.
**Step 8: Include proximal blobs with similar vectors:** We further expand the instance label generated in step 7 to include proximal blobs. For blobs not assigned to any instance labels, testing vectors are defined between the blob center and the blobs in the most recent instance label generated in step 7. Then we compute the angle between the testing vectors and the reference vector. The blob is then classified as part of the organelle instance label if any of the testing vectors satisfies the following two conditions: i) the absolute value of the cosine of the angle between the testing vector and the reference vector |*cos*(*θ*_*t*_*ref*_)| > *cos*(30°); ii) the length of the testing vector is shorter than the sum of the diameter of the two blobs in the testing vector (S2E and S2F Fig). Each time the organelle instance label is updated when adding a new blob. All non-assigned blobs proceed through the above classification again until no more blobs can be included in the instance label. Then we proceed to step 7 to find a new reference vector and initiate another instance label.
**Step 9: Cluster blobs with K-Means:** The remaining blobs not classified to any mitochondria instance label are clustered based on their coordinates by the K-Means clustering method [34]. K value is optimized according to the Elbow method [35]. Mitochondria instances without a reference vector are mostly of short length, close to an ellipsoidal morphology, which can be sufficiently represented by a few blobs. Thus, a K-Means clustering method can classify them. An example of K-Means clustering is shown in S3D Fig.
**Step 10: Translate information into scoring for ranking blobs:** So far, the instance labels have been generated based on blobs. But not all voxels in the semantic mask are included inside the blobs. A scoring function is computed for all voxels to determine if a voxel belongs to a specific blob. The function is defined as the distance between a voxel and a blob center divided by the radius of that blob. For each voxel, all blobs are ranked by the computed voxel scores.
**Step 11: Classify voxels to blobs:** Each voxel has multiple scores associated with it, one for each blob. The voxels from the semantic mask are assigned to the blob with the lowest score.
**Step 12: Classify blobs to instance:** Finally, all voxels in a blob are assigned with the organelle instance label of that blob.

#### Post-processing on a sphere-like organelle

The workflow to obtain instances of sphere-like organelle is indicated by the orange arrows in Fig 1. We use insulin vesicles as an example. With the input information including soft X-ray tomograms, insulin vesicle semantic segmentation masks, and the sphere-like morphology of insulin vesicle, we proceed through step 1 to step 6, then step 10 to step 12 to obtain insulin vesicle instance masks. For the sphere-like organelle, the instance label is the blob label.

#### Post-processing on a columnar-shaped organelle

The workflow to obtain instances of columnar-shaped organelle is indicated by the green arrows in Fig 1. We use mitochondria as an example. With the input information including soft X-ray tomograms, mitochondria semantic segmentation masks, and the columnar morphology of this mitochondrion, we proceed through step 1 to step 12 to obtain mitochondria instance mask. For the columnar-shaped organelle, the instance label might be one or several blobs.

### Validation

**Fig 2:**
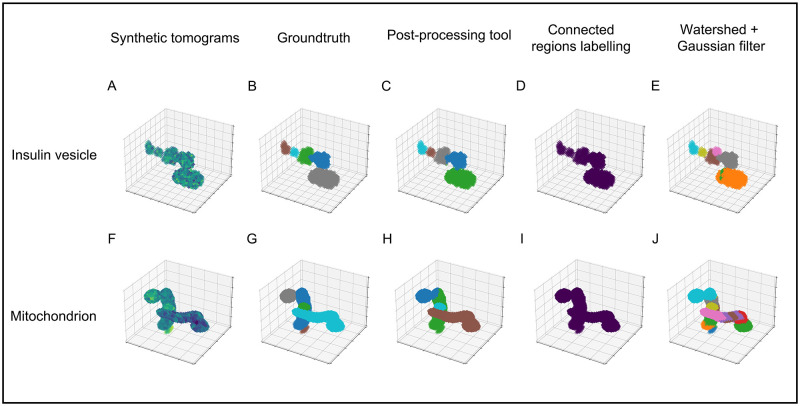
Separation results of mentioned methods on synthetic benchmarks. A) Synthetic tomogram of one insulin vesicle test dataset. Dark to light represents intensity from low to high. B) The groundtruth of insulin vesicle instance label, containing 5 instances. C) Results from post-processing tool, including 5 instances. D) Results from connected regions labeling method, including 1 instance. E) Results from watershed method, including 7 instances. Each color in images represents one insulin vesicle instance. F) Synthetic tomogram of one mitochondrion test dataset. Dark to light represents intensity from low to high. G) The groundtruth of mitochondria instance label, containing 5 instances. H) Results from post-processing tool, including 4 instances. I) Results from connected regions labeling method, including 1 instance. Each color in images represents one mitochondria instance. J) Results from watershed + Gaussian filter method, including 17 instances.

We apply our post-processing tool on synthetic tomograms (Materials and methods: Synthetic benchmark) to quantify the accuracy of the resulting organelle instances. We compare instances masks computed from our tool, connected regions labeling, Watershed methods (Materials and methods: Watershed Method), and watershed + Gaussian filter on insulin vesicles and mitochondria, respectively. The commonly used evaluation metric, Average Precision (AP) [36] is used here to measure the accuracy under different Intersection over Unions (IoUs, representing the similarity between the ground-truth and the predictions of individual organelle instances). We calculate the mean AP (mAP, AP@[.5:.95], obtained by averaging AP from AP50 to AP95 with 5% IoU increments) and AP in instance recognition under three different IoUs: 50% (AP50), 70% (AP70) and 90% (AP90), as listed in Table 1. To intuitively visualize the instance results, we plot the instance masks computed by three methods on one of the test datasets for each organelle type (Each dataset includes one synthetic tomogram and one semantic organelle mask): insulin vesicle (Fig 2A–2E) and mitochondrion (Fig 2F–2J).

**Table 1:** Instance mask AP on test datasets.

	Organelle	mAP(%)	AP50(%)	AP70(%)	AP90(%)
post-processing tool	insulin vesicle instance	**93.8**	**98.5**	**96.1**	**88.9**
connected regions labeling		1.0	10.0	0.0	0.0
watershed		42.6	53.9	48.1	29.9
watershed + Gaussian filter (*σ* = 1)		85.0	91.8	86.8	79.3
post-processing tool	mitochondrion instance	**49.9**	**68.3**	**53.4**	**36.5**
connected regions labeling		0.0	0.0	0.0	0.0
watershed		9.23	13.8	11.3	2.33
watershed + Gaussian filter (*σ* = 1)		35.4	46.4	40.9	18.7

All entries are average results from 10 test datasets.

As for insulin vesicle instance, 93.8% in mAP is obtained using our tool, whereas 85.0% in mAP is obtained using watershed + Gaussian filter and even lower mAPs using the other two methods. With an IoU threshold of 90%, our tool still results in 88.9%, whereas watershed + Gaussian filter shows only 29.9%. For example, one insulin vesicle instance (green, Fig 2B) is correctly separated using our tool (grey, Fig 2C). However, it is recognized as two instances using watershed + Gaussian filter (pink and brown, Fig 2E) due to the insignificant difference in the intensity between the edge of this insulin vesicle and the background (Fig 2A). Such uneven intensities are common in SXT, which might result from the uneven distribution of chemical components inside the insulin vesicle, or biases during SXT data collection [6].

Mitochondria instances (0.0%—49.9% in mAP) are generally more difficult to separate than insulin vesicle instances (1.0%—93.8% in mAP) for two reasons: 1) contacts between mitochondria increase the difficulty in classifying blobs at the edges (e.g., dark blue and green in Fig 2H, light blue and grey in Fig 2J); 2) overall low intensity of a mitochondrion leads to inaccurate blob detection (Fig 2F). Importantly, our tool still improves the accuracy of mitochondria instance results (49.9% in mAP) compared to the other three methods (35.4% or less in mAP). Thus, our post-processing tool provides a significantly improved accuracy than other commonly used instance separation methods by integrating prior knowledge of organelle morphology: the sphere-like shape of insulin vesicle and columnar-shaped of mitochondrion.

### Application

**Fig 3:**
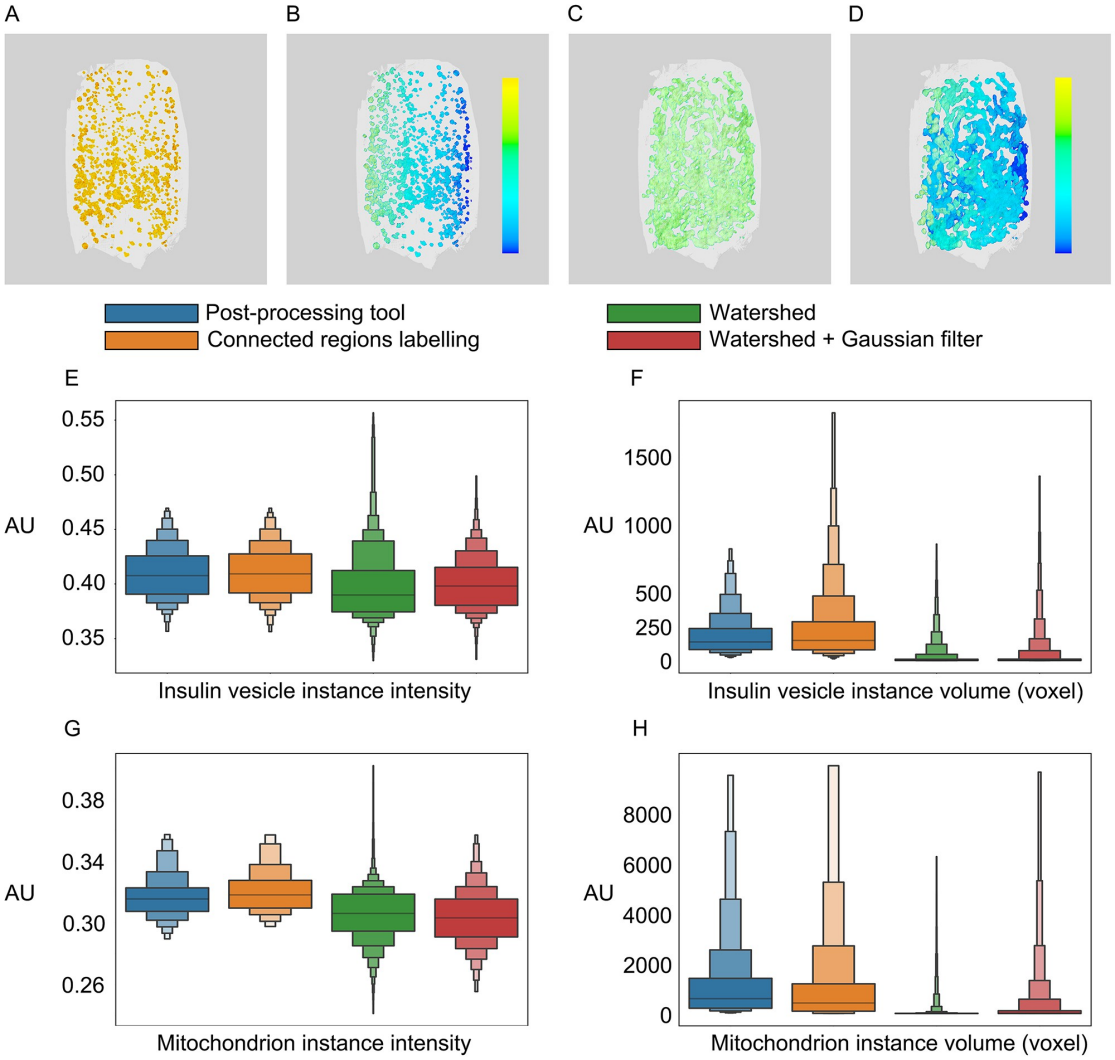
Results from post-processing tool on *β*-cell tomograms. The A) insulin vesicle semantic mask is processed into B) insulin vesicle instances mask, while the C) mitochondrion semantic mask is processed into D) mitochondria instances mask. Each color represents one organelle instance. E-H). Comparison of instance intensity and volume distribution from four mentioned methods in example datasets. AU: Arbitrary unit.

**Fig 4:**
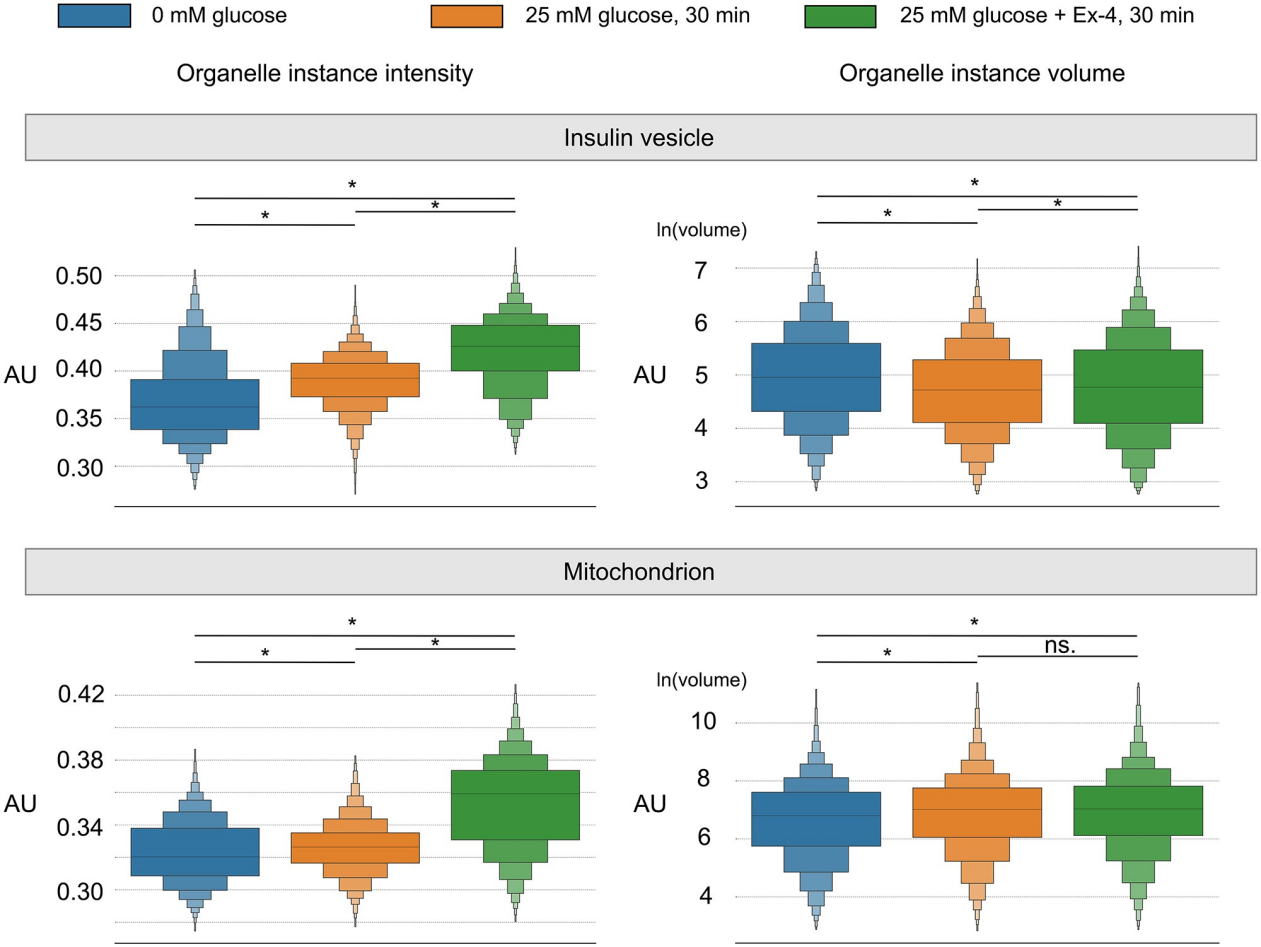
Analysis of insulin vesicle and mitochondria instances variance among conditions. Three conditions: 0 mM glucose, 25 mM glucose, 25 mM glucose + 10 nM Ex-4. Significance analysis are conveyed on Mann-whitney. *: p ≤1.0^−4^. AU: Arbitrary unit.

Insulin vesicles and mitochondria are organelles that play essential roles in insulin secretion. A single insulin vesicle refers to an insulin-containing dense-core secretory vesicle, which works as a cargo container to store, transport and secrete insulin [37]. A mitochondrion is a single mitochondrial unit which plays a role in providing ATP and triggering the plasma membrane depolarization during insulin secretion [38]. Here we apply our method on SXT tomograms of pancreatic *β*-cells and corresponding insulin vesicles and mitochondria masks (Materials and methods: Input data) (Fig 3A–3D). We first analyze the volume and intensity of individual organelles, and compare results from our tool with commonly used methods: connected regions labeling, watershed, and watershed + Gaussian filter method, as shown in Fig 3E–3H and S1 Table. Then, based on the intensity and volume for both insulin vesicle and mitochondria instances from our method, we investigate the impacts of glucose stimulation and Ex-4 on organelles in three conditions, as shown in Fig 4 and S2 Table.

We find that for insulin vesicles, the instance intensity distribution is similar between our method and the connected regions labeling method, but has wider ranges in the watershed and watershed + Gaussian filter methods (Fig 3E). In addition, the instance volume distribution is condensed from our method as compared with connected region labeling method, but is in the lower volume region as shown in the watershed and watershed + Gaussian filter methods, as shown in Fig 3F. Such distributions from the watershed and watershed + Gaussian filter methods are largely due to over-segmentation of instances. For the identification of mitochondrion, the distribution of a single mitochondrion’s intensity slightly shifts to low intensity region (Fig 3G) as compared with connected regions labeling. In contrast, the intensity distributions from watershed and watershed + Gaussian filter methods shift to even lower intensity regions. The volume of mitochondria instances shows similar trends as insulin vesicle instances: volume distribution is slightly condensed from our method (Fig 3H) compared with the connected regions labeling method, while the distributions from watershed and watershed + Gaussian filter methods are in lower volume regions. According to previous studies, the mean intensity of insulin vesicle instances in a cell ranges from 0.39 to 0.43 [2], while the average intensity of the whole mitochondria network is approximately 0.34 [2]. Our tool and connected regions labeling produce organelle instances which lie within the experimentally determined ranges, compared to Watershed and Watershed + Gaussian filer methods. The diameter of an insulin vesicle is approximately 200 nm [39–41], whose volume equals to 170 voxels in our tool results. The volume of mitochondrion ranges from 300 to 7500 voxels according to the baffle model [42]. Moreover, our tool produces organelle instances whose volumes agree well with the experimentally determined volume range compared to the other three methods. Such accordance in the organelle intensity and volumes further validates the performance of our tool.

Based on our method, we are able to detect the variance of individual organelles under different conditions to investigate the impacts of glucose stimulation and Ex-4 on the organelle, as shown in Fig 4. Results of intensity, volume, and number of organelle instances for each dataset are listed in S3 Table. For the glucose stimulation, compared with 0 mM glucose condition, insulin vesicle instances have increased intensity but decreased volume under 25 mM glucose condition. Previous studies have reported an insulin vesicle maturation process to form both small and large vesicles with different condensation levels of the content [43]. Thus, we suggest that glucose stimulation triggers *β*-cell to generate more small vesicles during the vesicle maturation. In addition, mitochondrion has increased intensity and volume with wider volume distribution under 25 mM glucose condition. A previous study noted increased heterogeneity of mitochondria under stimulation and diseased states [44], which resulted in larger variances in volume distribution. As for Ex-4, insulin vesicle instance intensity under 25 mM glucose + 10 nM Ex-4 condition is higher than the other two conditions, while the instance volume is higher than 25 mM glucose but lower than 0 mM glucose. Moreover, we observe that mitochondria instance intensity under 25 mM glucose + 10 nM Ex-4 condition is higher than the other two conditions, while the instance volume is higher than the 0 mM glucose condition, but has no difference compared to the 25 mM glucose condition. Ex-4 has been reported to enhance glucose-stimulated insulin secretion [45], but how Ex-4 affects the organelle molecular density during insulin secretion is still not clear. Our study provides extra morphological insights regarding the behavior of single insulin vesicle and mitochondria instances under different stimulation conditions; however, further biochemical studies are required to understand the priorities of single insulin vesicle and mitochondrion, as well as the interplay between individual organelles.

## Discussion

Segmentation is a critical step to identify a specific organelle from soft X-ray tomograms. However, current methods hardly satisfy our demands to obtain accurate organelle instances from tomograms. Thus, we propose a post-processing tool by integrating organelle intensities from SXT tomograms, organelle locations from semantic masks, and organelle morphology from prior knowledge. Specifically, our tool first identifies blobs in soft X-ray tomograms by finding the intensity local maxima points, then screens blobs by filtering with organelle semantic mask, third classifies those blobs to each organelle instance based on prior knowledge of organelle morphology.

In SXT tomograms, organelles like insulin vesicles, mitochondria, lipid droplets, and the nucleus have a higher LAC value (molecular density) than the cytosol surroundings. This feature allows for the identification of organelles by considering their LAC values and morphology [8, 46]. Our method identifies intensity local maxima points to separate organelle position directly from raw tomograms, improving our ability to distinguish individual organelles. In addition, our tool incorporates the prior knowledge of organelle morphology, making it possible to separate sphere-like organelle insulin vesicles and columnar-shaped organelle mitochondrion. For example, by using this tool, we can identify individual insulin vesicles that would often be segmented as one cluster in previous segmentation approaches, e.g. connected regions labeling. Given recent reports of insulin vesicle in contacts with other organelles [3] and increasing discussions on the relevance of inter-organelle contacts in cell biology [47–50], methods to accurately separate specific organelles will promote our understanding of such complex relationships. Another example is that we are able to identify individual mitochondrion using our tool, which is of grand challenge in previous segmentation approaches due to the columnar shape. Previous studies [2, 3, 50] have elegantly shown the complex mitochondria network but were not able to accurately distinguish between individual mitochondrion. Our approach will enable a detailed separation and characterization of individual mitochondria to better characterize how the organelle rearranges in health and disease. This approach will be beneficial for analyzing new data to compare the effects of disease states and drug treatments, specifically, the fragmentation of mitochondria network in diabetic *β*-cells [51] and glucagon-like peptide (GLP)-1 treatment on *β*-cells [52].

However, there are still opportunities to further improve our tool. First, user bias when manually segmenting semantic masks cannot be eliminated from the process. The final instance results will include mislabeled organelles from the semantic masks. Segmentation from multi-raters (multiple users identifying organelles) or using machine learning models will likely reduce such user bias. Second, prior knowledge of organelle morphology affects the accuracy of the final results. The representations of insulin vesicle in sphere-like shape and mitochondrion in columnar-shaped are based on current descriptions of from experiments [29, 42]. As separation methods develop, cases where insulin vesicles or mitochondria not existing in this specific morphology should be considered. Third, separation of organelles with more complex shapes, for example endoplasmic reticulum (ER) [53], remain a difficult task. To accurately separate the ER or Golgi body, new separation methods with a combination of different types of shapes are required. Fourth, the current implementation of our tool is time-consuming when calculating instance labels due to loops and iterations in the algorithm. Further improvements in algorithms are expected to improve the efficiency.

The validation of our approach in this manuscript reveals the potential to uncover new details on how cellular organization changes during specific cellular states or under specific drug treatments. Further use of this approach to investigate insulin vesicle organization will provide new insights into the functional maturation of insulin vesicles. It is understood that insulin vesicles exist in multiple functional pools such as the readily releasable pool (located near plasma membrane) and the reserve pool (located in the interior of the cell) [54]. Our results of insulin vesicle instances can be further exploited to investigate the reorganization of distinct pools during insulin secretion. In addition, mitochondria are activated [38] and driven to energy-consuming sites within cells [55] during the glucose-stimulated insulin secretion. Analysis of the individual mitochondrion obtained here will enable a more detailed investigation of the mitochondria localization. This will also allow for a more in-depth quantification of mitochondria organization depending on their location, inter-organelle contacts, and cellular states.

## Conclusion

In this paper, we proposed an intensity-based post-processing tool for separating organelle instances. Our tool incorporates information from soft X-ray tomograms, semantic masks, and prior knowledge of organelle morphology. We apply our tool on *β*-cells as a case study. Using this post-processing tool, we are able to separate sphere-like organelles, e.g., insulin vesicle instances, and columnar-shaped organelles, e.g., mitochondria instances. Results on synthetic benchmarks indicate that our tool has higher accuracy for analyzing both insulin vesicle and mitochondria instances than the commonly used separation methods such as connected regions labeling [28], watershed, and watershed + Gaussian filter [17]. In addition, we compare the instance results from all three methods on *β*-cell soft X-ray tomograms. We investigate the difference of instance intensity and volume on insulin vesicle and mitochondrion respectively. The results from our tool agree well with previous studies [39–42]. Moreover, with higher recognition accuracy, our tool reveals the significant variance of the volume and intensity of both insulin vesicle and mitochondria instances under different treatments, providing a more detailed description of the subcellular structures, which will is expected to facilitate future mechanistic studies of *β*-cells. Our tool can be further extended to separate other organelles in different cells, collected using multi-mode imaging techniques. Furthermore, our tool providing detailed descriptions of organelle will contribute to the efforts to increase precision and quality for whole-cell modeling [56–58].

## Supporting information

S1 Fig Filtered tomograms generated from an example tomogram applied with Gaussian filters with *σ* ranging from 1 to 10 with 1 increment. A-K) show the same slice of the example tomogram. The example tomogram is a region from *β*-cell dataset 822_4, including a cluster of organelles. Yellow in tomograms represents high intensity on that voxel, while dark purple represents the background. L) shows the corresponding semantic mask with blobs. Every red circle represents the center of a blob. Yellow in semantic mask L and raw tomogram K represent organelle label while dark purple represents the background.
(PDF)

S2 Fig Sketch of details from workflow. A) Example of fitting a sphere to an insulin vesicle. The full line represents the shape of the vesicle; the dotted line represents the fitted sphere on the vesicle. B) The evolution of overlapping ratio *a*_*r*_ along the fitted sphere radius. C) Calculation of the vectors from blob 2 towards its neighboring two blobs. D) The reference vector selected based on blob 2, marked green. Meanwhile, the three blobs are included in the same instance label. E) Generation of vectors from blob 4 to included blobs and compare the distance *d* and angle *α*. F) Blob 4 included in the instance label.
(PDF)

S3 Fig An example tomogram of a cluster of mitochondria. An example tomogram from *β*-cell dataset 822_4 shows a cluster of mitochondria with all blobs. A-D show the same slice of the 3D example tomogram. Each red circle is a 2D projection of the corresponding blob center from 3D spaces. A) Overview of all blobs on the mitochondrion cluster. B) One mitochondria instance label with five blobs generated from step 7 and step 8. C) Another mitochondria instance label with four blobs generated from step 7 and step 8. D) Last mitochondria instance label generated by K-Means clustering in step 9. The yellow circle represents the center of K-Means clustering for the three blobs. White in tomograms represents voxels with high intensity, while black represents background.
(PDF)

S1 Table Comparison of intensity and volume of insulin vesicle and mitochondria instances from four mentioned methods in example datasets. (PDF)

S2 Table Comparison of intensity and volume of insulin vesicle and mitochondria instances in three conditions. (PDF)

S3 Table Intensity, volume and instance number of insulin vesicle and mitochondria instance from all datasets. (PDF)
