# Supplementary material for: An intensity-based post-processing tool for 3D instance segmentation of organelles in soft X-ray tomograms
Source: PLoS One. 2022 Sep 1;17(9):e0269887. doi: 10.1371/journal.pone.0269887 (PMC9436087; doi:10.1371/journal.pone.0269887)
Supplement: S1 Fig — A-K) show the same slice of the example tomogram. The example tomogram is a region from β-cell dataset 822_4, including a cluster of organelles. Yellow in tomograms represents high intensity on that voxel, while dark purple represents the background. L) shows the corresponding semantic mask with blobs. Every red circle represents the center of a blob. Yellow in semantic mask L and raw tomogram K represent organelle label while dark purple represents the background. (PDF) [file pone.0269887.s001.pdf]

Supporting information

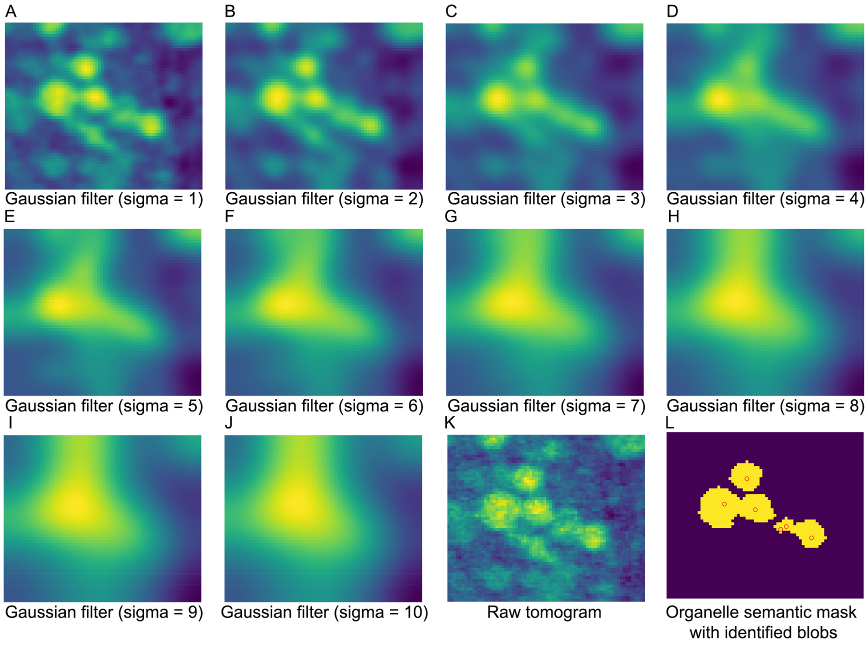

S1 Fig Filtered tomograms generated from an example tomogram applied with Gaussian filters with σ ranging from 1 to 10 with 1 increment. A-K) show the same slice of the example tomogram. The example tomogram is a region from β-cell dataset 822_4, including a cluster of organelles. Yellow in tomograms represents high intensity on that voxel, while dark purple represents the background. L) shows the corresponding semantic mask with blobs. Every red circle represents the center of a blob. Yellow in semantic mask L and raw tomogram K represent organelle label while dark purple represents the background.
