# Supplementary material for: An intensity-based post-processing tool for 3D instance segmentation of organelles in soft X-ray tomograms
Source: PLoS One. 2022 Sep 1;17(9):e0269887. doi: 10.1371/journal.pone.0269887 (PMC9436087; doi:10.1371/journal.pone.0269887)
Supplement: S2 Fig — A) Example of fitting a sphere to an insulin vesicle. The full line represents the shape of the vesicle; the dotted line represents the fitted sphere on the vesicle. B) The evolution of overlapping ratio ar along the fitted sphere radius. C) Calculation of the vectors from blob 2 towards its neighboring two blobs. D) The reference vector selected based on blob 2, marked green. Meanwhile, the three blobs are included in the same instance label. E) Generation of vectors from blob 4 to included blobs and compare the distance d and angle α. F) Blob 4 included in the instance label. (PDF) [file pone.0269887.s002.pdf]

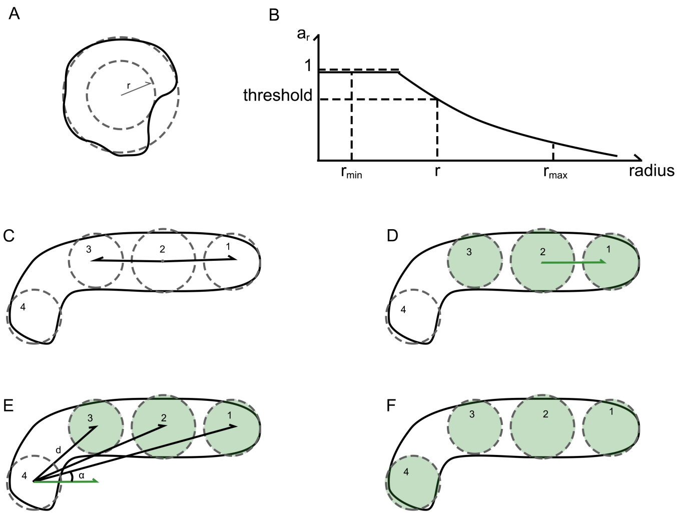

S2 Fig Sketch of details from workflow. A) Example of fitting a sphere to an insulin vesicle. The full line represents the shape of the vesicle; the dotted line represents the fitted sphere on the vesicle. B) The evolution of overlapping ratio a_r along the fitted sphere radius. C) Calculation of the vectors from blob 2 towards its neighboring two blobs. D) The reference vector selected based on blob 2, marked green. Meanwhile, the three blobs are included in the same instance label. E) Generation of vectors from blob 4 to included blobs and compare the distance d and angle α. F) Blob 4 included in the instance label.
