# Supplementary material for: An intensity-based post-processing tool for 3D instance segmentation of organelles in soft X-ray tomograms
Source: PLoS One. 2022 Sep 1;17(9):e0269887. doi: 10.1371/journal.pone.0269887 (PMC9436087; doi:10.1371/journal.pone.0269887)
Supplement: S3 Fig — An example tomogram from β-cell dataset 822_4 shows a cluster of mitochondria with all blobs. A-D show the same slice of the 3D example tomogram. Each red circle is a 2D projection of the corresponding blob center from 3D spaces. A) Overview of all blobs on the mitochondrion cluster. B) One mitochondria instance label with five blobs generated from step 7 and step 8. C) Another mitochondria instance label with four blobs generated from step 7 and step 8. D) Last mitochondria instance label generated by K-Means clustering in step 9. The yellow circle represents the center of K-Means clustering for the three blobs. White in tomograms represents voxels with high intensity, while black represents background. (PDF) [file pone.0269887.s003.pdf]

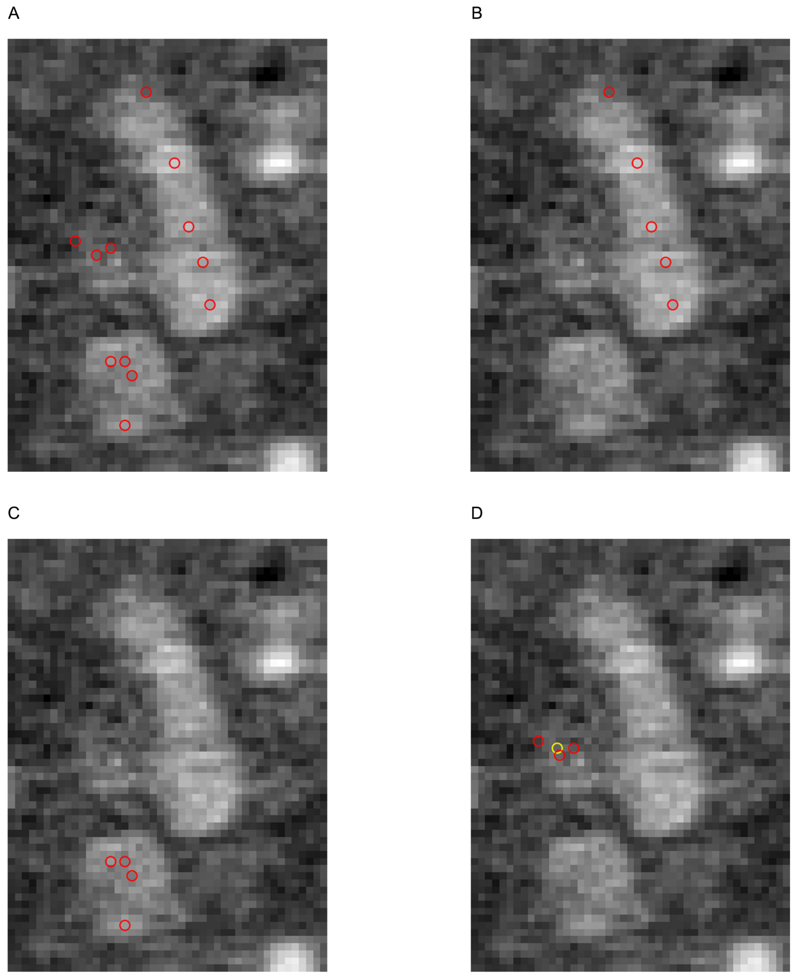

S3 Fig An example tomogram of a cluster of mitochondria. An example tomogram from β-cell dataset 822_4 shows a cluster of mitochondria with all blobs. A-D show the same slice of the 3D example tomogram. Each red circle is a 2D projection of the corresponding blob center from 3D spaces. A) Overview of all blobs on the mitochondrion cluster. B) One mitochondria instance label with five blobs generated from step 7 and step 8. C) Another mitochondria instance label with four blobs generated from step 7 and step 8. D) Last mitochondria instance label generated by K-Means clustering in step 9. The yellow circle represents the center of K-Means clustering for the three blobs. White in tomograms represents voxels with high intensity, while black represents background.
