# Supplementary material for: An intensity-based post-processing tool for 3D instance segmentation of organelles in soft X-ray tomograms
Source: PLoS One. 2022 Sep 1;17(9):e0269887. doi: 10.1371/journal.pone.0269887 (PMC9436087; doi:10.1371/journal.pone.0269887)
Supplement: S1 Table — (PDF) [file pone.0269887.s004.pdf]

S1 Table Comparison of intensity and volume of insulin vesicle and mitochondria instances from four mentioned methods in example datasets.

Organelle	Data type	Method	Mean	Standard deviation (SD)
Insulin vesicle instance	Intensity	Post-processing tool	0.409	0.025
		Connected region labelling	0.410	0.024
		Watershed	0.398	0.032
		Watershed + Gaussian filer	0.400	0.025
	Volume	Post-processing tool	190.593	166.759
		Connected region labelling	251.648	336.470
		Watershed	27.995	82.429
		Watershed + Gaussian filer	42.395	151.879
Mitochondria instance	Intensity	Post-processing tool	0.317	0.015
		Connected region labelling	0.320	0.016
		Watershed	0.305	0.017
		Watershed + Gaussian filer	0.304	0.019
	Volume	Post-processing tool	1353.319	2304.194
		Connected region labelling	1802.903	5653.956
		Watershed	50.650	330.936
		Watershed + Gaussian filer	443.766	2307.269

Dataset 769_5 for insulin vesicle and 783_5 for mitochondria results.
