# Supplementary material for: An intensity-based post-processing tool for 3D instance segmentation of organelles in soft X-ray tomograms
Source: PLoS One. 2022 Sep 1;17(9):e0269887. doi: 10.1371/journal.pone.0269887 (PMC9436087; doi:10.1371/journal.pone.0269887)
Supplement: S2 Table — (PDF) [file pone.0269887.s005.pdf]

S2 Table Comparison of intensity and volume of insulin vesicle and mitochondria instances in three conditions.

Organelle	Data type	Condition	Mean	Standard error (SE)
Insulin vesicle instance	Intensity	0 mM glucose	0.368	0.016
		25 mM glucose	0.389	0.011
		25 mM glucose + 10 nM Ex-4	0.421	0.014
	ln(volume)	0 mM glucose	4.957	0.343
		25 mM glucose	4.702	0.315
		25 mM glucose + 10 nM Ex-4	4.771	0.354
Mitochondria instance	Intensity	0 mM glucose	0.323	0.008
		25 mM glucose	0.326	0.006
		25 mM glucose + 10 nM Ex-4	0.353	0.010
	ln(volume)	0 mM glucose	6.645	0.536
		25 mM glucose	6.865	0.525
		25 mM glucose + 10 nM Ex-4	6.918	0.537
