# Supplementary material for: An intensity-based post-processing tool for 3D instance segmentation of organelles in soft X-ray tomograms
Source: PLoS One. 2022 Sep 1;17(9):e0269887. doi: 10.1371/journal.pone.0269887 (PMC9436087; doi:10.1371/journal.pone.0269887)
Supplement: S3 Table — (PDF) [file pone.0269887.s006.pdf]

S3 Table Intensity, volume and instance number of insulin vesicle and mitochondria instance from all datasets.

dataset index	condition	insulin vesicle instance			mitochondria instance		
		intensity	volume (voxel)	number	intensity	volume (voxel)	number
		Mean ± SD	Mean ± SD		Mean ± SD	Mean ± SD	
783_5	0 mM glucose	0.366 ± 0.017	198.788 ± 220.883	424	0.317 ± 0.015	1353.319 ± 2304.194	329
783_11		0.427 ± 0.032	458.053 ± 363.230	246	0.338 ± 0.014	1993.366 ± 6177.615	235
783_12		0.339 ± 0.040	189.587 ± 160.486	419	0.303 ± 0.010	3614.815 ± 10641.954	233
784_4		0.410 ± 0.040	245.661 ± 219.266	519	0.356 ± 0.010	3325.519 ± 15752.424	212
784_5		0.361 ± 0.025	88.133 ± 94.789	330	0.343 ± 0.010	1448.872 ± 3280.561	235
784_6		0.349 ± 0.027	128.731 ± 95.413	201	0.317 ± 0.014	2066.881 ± 7869.161	396
784_7		0.335 ± 0.022	235.514 ± 208.655	321	0.307 ± 0.013	1636.410 ± 3293.909	310
785_7		0.355 ± 0.024	190.442 ± 165.586	400	0.320 ± 0.014	1928.132 ± 6617.971	468
766_2	25 mM glucose	0.392 ± 0.028	182.439 ± 176.529	652	0.348 ± 0.012	1522.861 ± 2749.621	252
766_5		0.407 ± 0.020	172.650 ± 163.104	1127	0.327 ± 0.01	2920.504 ± 9277.321	260
766_7		0.392 ± 0.024	118.058 ± 110.957	640	0.320 ± 0.014	2261.959 ± 3435.018	244
766_8		0.355 ± 0.031	167.971 ± 199.826	943	0.301 ± 0.011	4338.274 ± 11320.585	241
766_10		0.393 ± 0.020	176.732 ± 157.087	1153	0.330 ± 0.014	1915.149 ± 6934.459	484
766_11		0.376 ± 0.022	115.605 ± 101.687	1337	0.321 ± 0.013	1912.473 ± 3276.707	421
769_5		0.409 ± 0.025	190.593 ± 166.759	945	0.332 ± 0.012	2133.130 ± 6576.227	262
769_7		0.397 ± 0.018	123.969 ± 114.712	830	0.327 ± 0.008	5053.737 ± 15091.523	205
822_4	25 mM glucose + 10 nM Ex-4	0.437 ± 0.021	244.278 ± 218.582	900	0.368 ± 0.017	3028.817 ± 8864.580	327
822_6		0.447 ± 0.024	199.617 ± 158.984	674	0.365 ± 0.018	2111.572 ± 2412.439	360
822_7		0.422 ± 0.032	149.896 ± 153.057	797	0.360 ± 0.019	2386.053 ± 4019.660	378
842_12		0.349 ± 0.017	146.882 ± 175.937	516	0.304 ± 0.014	5376.675 ± 24874.676	163
842_13		0.400 ± 0.025	270.933 ± 227.049	418	0.326 ± 0.013	2425.066 ± 7948.511	256
842_17		0.414 ± 0.022	219.955 ± 197.866	379	0.323 ± 0.016	3875.055 ± 15028.223	237
931_9		0.436 ± 0.027	108.525 ± 110.226	413	0.377 ± 0.016	2520.682 ± 5733.090	236
931_14		0.438 ± 0.027	91.663 ± 141.772	570	0.374 ± 0.014	2805.640 ± 9085.129	261

SD: standard deviation.
